# Clinical, biochemical, and molecular genetic characteristics of patients with primary carnitine deficiency identified by newborn screening in Shanghai, China

**DOI:** 10.3389/fgene.2022.1062715

**Published:** 2022-12-08

**Authors:** Siyu Chang, Yi Yang, Feng Xu, Wenjun Ji, Xia Zhan, Xiaolan Gao, Ting Chen, Wenjuan Qiu, Huiwen Zhang, Lili Liang, Deyun Lu, Kaichuang Zhang, Xuefan Gu, Lianshu Han

**Affiliations:** Department of Pediatric Endocrinology and Genetic Metabolism, Shanghai Institute for Pediatric Research, Xinhua Hospital, Shanghai Jiaotong University School of Medicine, Shanghai, China

**Keywords:** primary carnitine deficiency, SLC22A5, newborn screening, tandem mass spectrometry, free carnitine

## Abstract

**Background:** Primary carnitine deficiency (PCD) is an autosomal recessive disease caused by mutations in the *SLC22A5* gene, which encodes the organic cation transporter 2 (OCTN2). Patients with PCD may be at risk of skeletal or cardiac myopathy, metabolic decompensation, and even sudden death. This study aimed to analyze the biochemical, clinical, and genetic characteristics of PCD patients identified by newborn screening (NBS) in Shanghai.

**Methods:** Dried blood spot (DBS) samples of newborns were analyzed through tandem mass spectrometry (MS/MS) from January 2003 to December 2021. Newborns with low free carnitine (C0) levels were recalled. Mutation in the *SLC22A5* gene was analyzed on suspected positive newborns with low C0 levels after recall.

**Results:** 1,247,274 newborns were screened by MS/MS and 40 newborns were diagnosed with PCD, therefore the incidence of PCD in Shanghai was approximately 1:31,200. The mean C0 level in newborns with PCD was 5.37 ± 1.79 μmol/L before treatment and increased to 24.45 ± 10.87 μmol/L after treatment with L-carnitine. Twenty-three different variants were identified in the *SLC22A5* gene, including 8 novel variants, of which c.51C>G (p.F17L) was the most frequent (27.27%, 18/66), followed by c.1400C>G (p.S467C) (25.76%, 17/66). Almost all the screened PCD patients were asymptomatic.

**Conclusion:** NBS *via* MS/MS was a quick and efficient method for the early diagnosis of PCD. The incidence of PCD in Shanghai was 1:31,200. Eight novel variants were identified, which greatly expanded the variant spectrum of *SLC22A5*. MS/MS combined with genetic testing could effectively improve the diagnostic accuracy of PCD.

## Introduction

Primary carnitine deficiency (PCD, OMIM 212140) refers to an autosomal recessive disorder of fatty acid oxidation caused by mutations in the *SLC22A5* gene, which encodes the organic cation transporter 2 (OCTN2) (Tang et al., 1999; Cederbaum et al., 2002). The *SLC22A5* gene is located on the chromosome 5q31.1 and contains over 150 reported disease-causing variants (Zhang et al., 2019). The incidence of PCD varies in different countries, which is estimated to be 1:142,000 in the United States, 1:40,000 in Japan, and even 1:300 in the Faroe Islands (Koizumi et al., 1999; Rasmussen et al., 2014; Therrell et al., 2014). Similarly, it varies in different regions throughout the China, ranging from 1:9,000 to 1:34,000 (Sun et al., 2017; Zhou et al., 2019; Lin et al., 2020; Lin et al., 2021). The clinical manifestations of PCD are diverse, mainly manifested as metabolic disorders, myocardial and skeletal muscle damage in infancy and early childhood, or muscle weakness and arrhythmia in adult (Saudubray and Garcia-Cazorla 2018). Patients with PCD may be at risk of sudden death due to cardiac failure (Longo et al., 2006; Kepka et al., 2011). Fortunately, early diagnosis by newborn screening (NBS) *via* tandem mass spectrometry (MS/MS) technology and timely supplementation of l-carnitine in the neonatal period can effectively prevent the poor outcome (Longo et al., 2016).

The main objective of this study was to analyze the clinical, biochemical, and molecular genetic characteristics of PCD patients identified by NBS in Shanghai. In this work, over 18-year experience of NBS for PCD was summarized and the genotype characteristics and clinical phenotype of the patients were compared, aiming to provide meaningful information for the large-scale screening and variant spectrum of *SLC22A5* gene.

## Patients and methods

### Research subjects

NBS *via* MS/MS was first introduced in China in 2003 at Xinhua Hospital, Shanghai Jiaotong University School of Medicine. From January 2003 to December 2021, 1,247,274 newborns were recruited for PCD screening in our hospital, accounting for approximately 70% of all infants born in Shanghai. This study was approved by the Ethical Committee of Xinhua Hospital, Shanghai Jiaotong University School of Medicine (Approval No. XHEC-D-2021-139). The written informed consent was obtained from the parents or guardians of newborns.

### NBS for PCD

Neonatal blood samples collected from heels were spotted on the filter papers from various maternity hospitals in Shanghai within 72 h to 7 days after birth. Dried blood spot (DBS) samples were sent to Xinhua Hospital Newborn Screening Center by cold-chain transportation after natural air drying. DBS samples were pre-processed using a derivatized method and analyzed by Micromass Quattro micro API Mass spectrometer (2000, 4000 or 4500). Sample preparation and MS/MS analysis strictly followed the laboratory standard operating procedures. The cut-off value of C0 in our laboratory was 10–60 μmol/L (Han et al., 2014). Newborns with low C0 levels and their mothers were recalled to collect the DBS samples for MS/MS detection. Subsequently, the mutations in *SLC22A5* gene were analyzed on suspected positive newborns to further confirm the diagnosis.

### Genetic analysis

Genomic DNA of suspected positive newborns and their parents was extracted from peripheral venous blood, primary screening or recalled DBS samples by using a blood DNA isolation kit (Tiangen Biotech Co. Ltd.). The 10 exons and their boundaries of the *SLC22A5* gene were amplified by polymerase chain reaction and analyzed on an automated DNA sequencer (ABI3700, Applied Biosystems). The mutations in *SLC22A5* gene were analyzed on suspected PCD patients using the Sanger sequencing. The mutations were identified using a normal human *SLC22A5* sequence as a reference (NM_003060.4), and the gene sequencing results were compared with the data in the HGMD, LOVD, ClinVar, and dbSNP databases to obtain information about the pathogenic mutation sites. The DNA samples of their parents were used to verify if the mutation originated from the father or mother. The possible pathogenicity of novel variants was evaluated by using the PolyPhen-2, SIFT, LRT, Mutation Taster PROVEAN and GERP++. The effects of novel missense mutations on protein functions were predicted by HOPE (http://www.cmbi.ru.nl/hope/; Venselaar et al., 2010).

### Diagnostic criteria

Patients were diagnosed with PCD based on C0 levels, genetic mutations, and clinical symptoms. Newborns with two variants in the *SLC22A5* gene were diagnosed with PCD. The suspected positive newborns not performing genetic testing or carrying one variant after exclusion of maternal and secondary carnitine deficiencies but with consecutively lower C0 levels until treatment were diagnosed with PCD.

### Treatment and follow-up

PCD patients were treated with l-carnitine supplementation as soon as it was diagnosed. Generally, the dosage of oral administration is 50–200 mg/kg/day, divided into three times a day. The dosage of l-carnitine supplementation was adjusted according to different C0 levels in individuals. PCD patients were followed up monthly during the initial treatment period and then once every 2–3 months after the C0 level was normal and stable. Other regular follow-up items included blood ammonia, blood sugar, liver function, creatine kinase, abdominal ultrasound, electrocardiogram, echocardiography, growth, and intelligence development assessment.

### Statistical analysis

SPSS 16.0 (SPSS Inc., Chicago, IL, United States) was adopted for statistical analysis. The Student’s t-test was applied to compare the difference of C0 levels between two groups, and *p* < 0.05 meant the difference was statistically significant.

## Results

### NBS for PCD

1,247,274 newborns were screened by MS/MS, and 984 infants primarily displayed low C0 levels, so the positive rate was 0.08%. Among them, 934 newborns were successfully recalled, and the positive recall rate was 95%. Eventually, 40 newborns including 18 males and 22 females were diagnosed with PCD, yielding a positive predictive value (PPV) of 4.28% (40/934). In addition, 15 newborns were diagnosed with maternal PCD. From these results, the incidence of PCD in Shanghai was approximately 1:31,200.

### Biochemical characteristics

**FIGURE 1:**
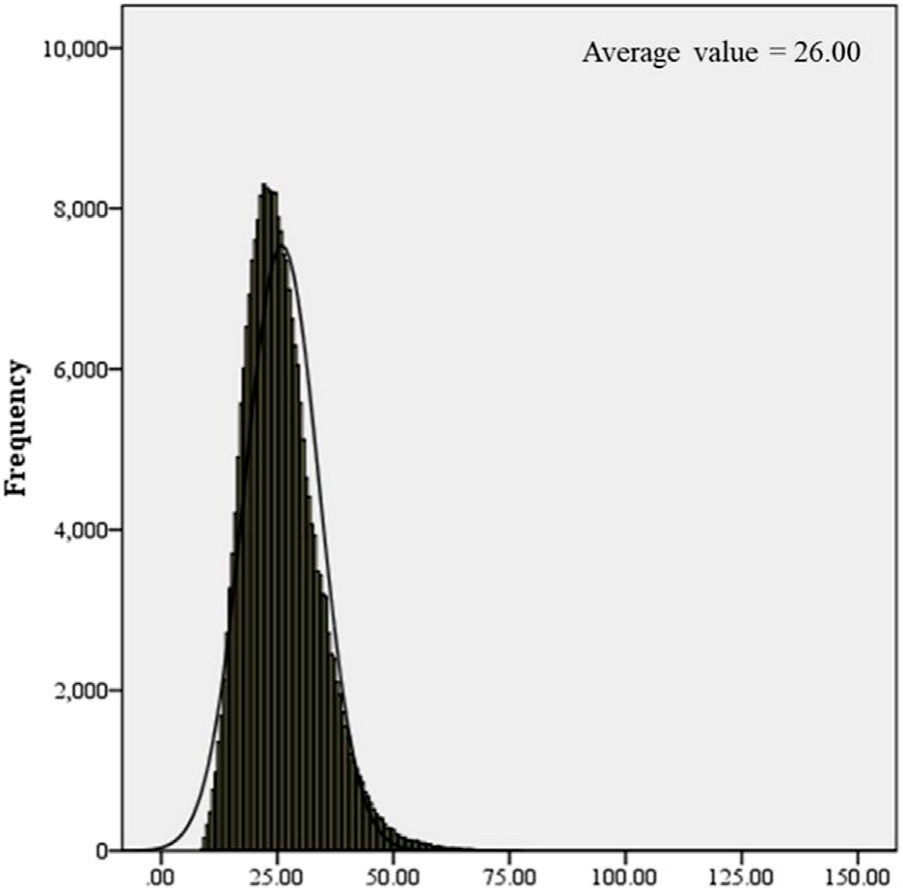
Frequency distribution histogram of C0.

**FIGURE 2:**
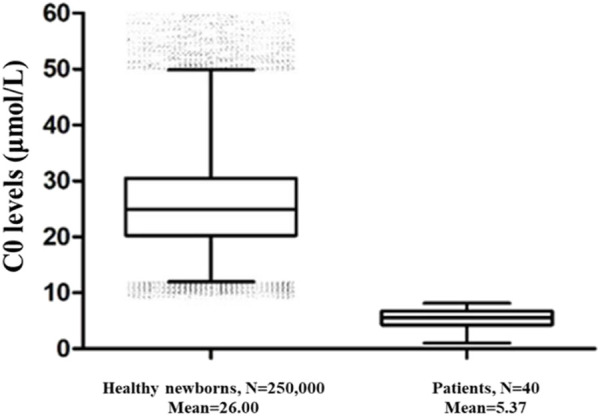
The C0 levels in healthy newborns and PCD patients. 250,000 healthy newborns were randomly selected for comparative analysis. The error bars are 1th and 99th percentiles.

250,000 healthy newborns were randomly selected to describe the frequency distribution of C0 levels. C0 levels in the DBS samples from healthy newborns exhibited a normal distribution, with the 0.1%–99.9% and 0.5%–99.5% confidence intervals (CIs) of 9.84–62.52 μmol/L and 11.09–54.10 μmol/L, respectively (Figure 1). The C0 level in healthy newborns was 26.00 ± 8.01 μmol/L (mean ± standard deviation) (Figure 2). All newborns and mothers with PCD presented decreased C0 levels. C0 levels of PCD newborns at the primary screening and after the recall were 5.37 ± 1.79 μmol/L and 5.57 ± 2.13 μmol/L, respectively (Figure 2). The C0 level in mothers with PCD was 3.05 ± 1.28 μmol/L, and that in newborns with PCD was 20% of that in healthy newborns.

### Clinical presentation and follow up

PCD patients were treated with l-carnitine supplementation immediately after diagnosis, and the median age of patients at the initial treatment was 30 days. After treatment for about 1 month, the C0 level (24.45 ± 10.87 µmol) was significantly higher than that in primary screening (*t* = 9.668, *p* < 0.001). The follow-up period of PCD patients ranged from 1 month to 16 years, and the C0 level in most patients remained normal and stable. Case 3 claimed to be not good at sports. Case 15 was treated with l-carnitine supplementation since diagnosis to maintain the normal and stable C0 level, and suffered from easy fatigue and mild left ventricular enlargement. Case 17 was treated with l-carnitine supplementation for a short time after birth and then stopped treatment privately for 5 years; the C0 level in the most recent followed-up was 3.38 μmol/L, and muscle strength was weakness. The remaining PCD patients followed-up in our hospital were asymptomatic, with normal growth and development, no apparent abnormalities of heart and lung, and with good limb activity.

### *SLC22A5* gene mutation analysis

Thirty-six infants identified as having low C0 levels underwent *SLC22A5* gene mutation analysis. Thirty newborns with PCD harbored two variants in *SLC22A5*, and six had one variant (Table 1). Twenty-three different variants were identified in *SLC22A5* gene from 66 mutant alleles. Among them, 69.57% (16/23) were missense mutations, 17.39% (4/23) were non-sense mutations, 4.35% (1/23) were splicing mutations, and 8.70% (2/23) were frameshift mutations. Fifteen variants have been previously reported in the HGMD, LOVD, ClinVar, and dbSNP databases, and another eight were firstly found. Twelve variants were reported as pathogenic or likely pathogenic mutations, one variant is of uncertain clinical significance, and the remaining variants have no clinical classification (Table 2). The possible pathogenicity of 8 novel variants analyzed by PolyPhen-2, SIFT, LRT, Mutation Taster, PROVEAN, or GERP++ was listed in Table 3. The Sanger sequencing results of eight new mutations of *SLC22A5* gene in PCD newborns and their parents was shown in Supplementary Figure S1. The mean primary screening and recalled C0 levels in four patients with c.51C>G (p.F17L) and a non-sense mutation or frameshift mutation (4.15 ± 0.67 μmol/L and 3.12 ± 0.58 μmol/L, respectively) were lower than those of 11 patients with c.51C>G (p.F17L) and missense mutation (5.92 ± 1.39 μmol/L and 6.55 ± 2.06 μmol/L, respectively), and the differences were statistically significant (t = 2.39, two-tailed *p*-value = 0.032, and t = 3.23, two-tailed *p*-value = 0.006, respectively).

**TABLE 1:** Clinical, biochemical, and molecular characteristics of PCD patients identified by NBS.

Case	Sex	GW	Birth weight (kg)	Initial treatment age	Current age (y)	Symptoms	C0 levels, µmol/L			*SLC22A5* gene mutation	
							Primary screening	Recall	After treatment	Allele 1	Allele 2
1	M	39	2.80	4y	16	Loss of follow-up	5.32	7.30	13.77	c.51C>G (p.F17L)	c.797C>T (p.P266L)
2	F	38	3.27	35d	16	Loss of follow-up	3.24	3.18	17.98	c.51C>G (p.F17L)	c.248G>T (p.R83L)
3	M	38 + 3	3.31	37d	13	Asymptomatic	1.03	3.63	11.33	c.396G>A (p.W132X)	c.1400C>G (p.S467C)
4	F	39 + 6	3.06	20d	12	Asymptomatic	4.28	4.23	36.98	c.51C>G (p.F17L)	c.1195C>T (p.R399W)
5	M	40 + 1	3.73	54d	11	Asymptomatic	4.94	8.00	36.99	c.338G>A (p.C113Y)	c.797C>T (p.P266L)
6	F	37 + 1	2.46	44d	11	Asymptomatic	5.16	6.38	17.53	c.51C>G (p.F17L)	c.1133T>G (p.L378R)
7	M	39	3.53	2m	10	Asymptomatic	7.42	7.63	9.08	c.428C>T (p.P143L)	c.1400C>G (p.S467C)
8	F	40 + 3	3.13	14d	10	Loss of follow-up	6.30	4.34	-	c.51C>G (p.F17L)	c.1400C>G (p.S467C)
9	M	38 + 2	3.05	30d	10	Asymptomatic	2.13	1.85	47.61	c.1064C>T (p.S355L)	Not detected
10	F	40	3.00	46d	9	Loss of follow-up	3.31	2.85	-	c.51C>G (p.F17L)	c.384dupT (p.V129Cfs*9)
11	M	40	3.44	28d	9	Asymptomatic	4.01	3.10	18.68	c.51C>G (p.F17L)	c.361C>T (p.Q121X)
12	F	38 + 1	3.27	27d	8	Asymptomatic	7.85	6.34	22.73	c.428C>T (p.P143L)	c.1400C>G (p.S467C)
13	M	39 + 2	3.23	-	8	Asymptomatic	2.31	4.14	-	c.51C>G (p.F17L)	c.51C>G (p.F17L)
14	F	39 + 6	3.22	21d	7	Loss of follow-up	4.72	5.18	-	c.338G>A (p.C113Y)	c.1400C>G (p.S467C)
15	M	39 + 2	3.70	22d	7	Easy fatigue	7.56	6.15	20.08	c.51C>G (p.F17L)	c.1400C>G (p.S467C)
						Cardiomyopathy					
16	F	38 + 5	3.32	45d	6	Asymptomatic	7.03	6.47	20.24	c.1400C>G (p.S467C)	Not detected
17	M	38	3.30	35d	6	Muscle weakness	6.32	8.83	15.78	c.51C>G (p.F17L)	c.1445A>G (p.Y482C)
18	F	39 + 3	3.70	-	5	Loss of follow-up	8.12	9.87	-	c.51C>G (p.F17L)	c.1400C>G (p.S467C)
19	F	40 + 3	4.26	18d	5	Asymptomatic	6.26	7.16	20.96	c.51C>G (p.F17L)	c.797C>T (p.P266L)
20	F	40 + 6	3.75	-	5	Loss of follow-up	6.70	6.69	-	c.760C>T (p.R254X)	Not detected
21	F	38 + 3	2.91	-	5	Loss of follow-up	2.20	4.99	-	c.254_264dup (p.I89Gfs*45)	Not detected
22	M	38 + 4	3.93	20d	4	Asymptomatic	6.40	4.46	26.61	c.51C>G (p.F17L)	Not detected
23	F	39 + 2	2.96	40d	4	Asymptomatic	7.30	5.77	24.22	c.497 + 1G>T	c.680G>A (p.R227H)
24	M	37 + 5	3.34	23d	4	Asymptomatic	5.84	9.16	31.03	c.51C>G (p.F17L)	c.1400C>G (p.S467C)
25	M	38 + 1	3.27	20d	4	Asymptomatic	4.40	3.92	15.73	c.51C>G (p.F17L)	c.760C>T (p.R254X)
26	F	39	2.56	32d	4	Asymptomatic	5.70	6.06	-	c.760C>T (p.R254X)	c.1400C>G (p.S467C)
27	F	39 + 3	3.54	3m	3	Asymptomatic	4.60	3.67	22.30	c.407G>A (p.C136Y)	c.1400C>G (p.S467C)
28	F	38 + 5	3.70	31d	3	Asymptomatic	5.00	1.87	28.51	c.248G>A (p.R83H)	c.248G>A (p.R83H)
29	M	39 + 3	4.07	35d	3	Asymptomatic	7.20	9.30	30.74	c.1199G>A (p.R400H)	c.1400C>G (p.S467C)
30	F	41 + 1	3.84	24d	3	Asymptomatic	4.90	2.26	26.41	c.51C>G (p.F17L)	c.844C>T (p.R282X)
31	F	40 + 4	4.04	27d	2	Asymptomatic	6.20	7.05	15.10	c.797C>T (p.P266L)	Not detected
32	F	35 + 2	1.98	28d	1	Asymptomatic	5.20	5.03	21.05	c.621G>T (p.Q207H)	c.760C>T (p.R254X)
33	F	37 + 4	3.20	30d	1	Asymptomatic	6.70	4.82	47.49	c.338G>A (p.C113Y)	c.1400C>G (p.S467C)
34	M	38 + 6	3.29	18d	1	Asymptomatic	6.70	5.88	36.45	c.51C>G (p.F17L)	c.1400C>G (p.S467C)
35	F	40 + 3	3.27	21d	1	Asymptomatic	6.40	6.65	38.72	c.1400C>G (p.S467C)	c.1400C>G (p.S467C)
36	M	39	3.55	36d	1	Asymptomatic	7.60	4.52	33.17	c.494A>G (p.D165G)	c.1400C>G (p.S467C)

F, female; M, male; GW, gestational weeks; d, days; y, years.

**TABLE 2:** Frequencies, locations, and clinical classification of detected mutations.

NO	Mutations	Exons	Mutant allele (NO.)	Frequency (%)	ClinVar (clinical classification)	References
1	c.51C>G (p.F17L)	Exon 1	18	27.27	P/LP	(Wang et al., 1999)
2	c.248G>T (p.R83L)	Exon 1	1	1.52	P/LP	(Wang et al., 1999)
3	**c.248G>A (p.R83H)**	Exon 1	2	3.03	N.F	This study
4	c.254_264dup (p.I89Gfs*45)	Exon 1	1	1.52	P	[Wang et al., 2001]
5	c.338G>A (p.C113Y)	Exon 1	3	4.55	P/LP	(Han et al., 2014)
6	**c.361C>T (p.Q121X)**	Exon 1	1	1.52	N.F	This study
7	c.384dupT (p.V129Cfs*9)	Exon 1	1	1.52	N.F	This study
8	c.396G>A (p.W132X)	Exon 2	1	1.52	P	(Koizumi et al., 1999)
9	**c.407G>A (p.C136Y)**	Exon 2	1	1.52	N.F	This study
10	c.428C>T (p.P143L)	Exon 2	2	3.03	LP	(Lee et al., 2010)
11	**c.494A>G (p.D165G)**	Exon 2	1	1.52	N.F	This study
12	c.497 + 1G>T	Intron 2	1	1.52	N.F	(Han et al., 2014)
13	**c.621G>T (p.Q207H)**	Exon 3	1	1.52	N.F	This study
14	c.680G>A (p.R227H)	Exon 4	1	1.52	LP	(Li et al., 2010)
15	c.760C>T (p.R254X)	Exon 4	4	6.06	P	(Tang et al., 2002)
16	c.797C>T (p.P266L)	Exon 4	4	6.06	P	(Han et al., 2014)
17	c.844C>T (p.R282X)	Exon 5	1	1.52	P	(Wang et al., 1999)
18	c.1064C>T (p.S355L)	Exon 7	1	1.52	VUS	(Li et al., 2010)
19	**c.1133T>G (p.L378R)**	Exon 7	1	1.52	N.F	This study
20	c.1195C>T (p.R399W)	Exon 7	1	1.52	P/LP	(Wang et al., 1999)
21	**c.1199G>A (p.R400H)**	Exon 7	1	1.52	N.F	This study
22	c.1400C>G (p.S467C)	Exon 8	17	25.76	P/LP	(Koizumi et al., 1999)
23	c.1445A>G (p.Y482C)	Exon 8	1	1.52	N.F	(Lin et al., 2020)

P, pathogenic; LP, likely pathogenic; N.F, not found; VUS, uncertain clinical significance.

The novel variants of this study are shown in boldface type.

**TABLE 3:** List of the 8 novel variants and their possible pathogenicity analysis.

	Mutations	PolyPhen-2	SIFT	LRT	Mutation taster	PROVEAN	GERP++
1	c.248G>A (p.R83H)	Probably-damaging	Damaging	Deleterious	Disease-causing	Damaging	Conserved
2	c.361C>T (p.Q121X)	-	-	Neutral	Disease-causing	-	Conserved
3	c.384dupT (p.V129Cfs*9)	-	-	-	Disease-causing	-	-
4	c.407G>A (p.C136Y)	Probably-damaging	Damaging	Deleterious	Disease-causing	Damaging	Conserved
5	c.494A>G (p.D165G)	Possibly-damaging	Tolerable	Deleterious	Disease-causing	Damaging	Conserved
6	c.621G>T (p.Q207H)	Benign	Tolerable	Deleterious	Disease-causing	Damaging	Conserved
7	c.1133T>G (p.L378R)	Probably-damaging	Damaging	Deleterious	Disease-causing	Damaging	Conserved
8	c.1199G>A (p.R400H)	Possibly-damaging	Tolerable	Deleterious	Disease-causing	Damaging	Conserved

The most common mutation in PCD newborns was c.51C>G (p.F17L) with a frequency of 27.27% (18/66), followed by c.1400C>G (p.S467C) (25.76% (17/66)). In addition, c.760C>T (p.R254X) (6.06%), c.797C>T (p.P266L) (6.06%), and c.338G>A (p.C113Y) (4.55%) were relatively common. Almost all the identified variants were scattered on exons except one on intron.

The effects of protein structure and function on the 6 novel missense mutations were predicted by HOPE, and the diagram of 3D-protein structure was shown in Supplementary Figure S2. 1) p. R83H: the mutant residue was smaller than the wild-type Arg 83 residue. This difference in size might lead to loss of interactions. 2) p. C136Y: the mutant residue was bigger than the wild-type Cys 136 residue, which might lead to bumps. The mutant residue was located near a highly conserved position and probably damaged the protein. 3) p. D165G: the mutation introduced a very flexible glycine at position 165, which can disturb the required rigidity of the protein. The domain of mutant residue located was important for binding with other molecules. The charge of the wild-type Asp 165 residue was lost due to this mutation, which can cause loss of interactions with other molecules. 4) p. Q207H: the wild-type Glu 207 residue and mutant residue differ in size. The mutant residue was bigger than the wild-type residue, which can affect the contact with the lipid-membrane. 5) p. L378R: the mutant residue was bigger than the wild-type Leu 378 residue and could affect the contact with the lipid-membrane. The wild-type residue was more hydrophobic than the mutation, which might result in the loss of hydrophobic interactions with other molecules on the protein surface. 6) p. R400H: the mutant residue was smaller than the wild-type residue, and the charge of the wild-type residue was lost by this mutation, which might cause a possible loss of external interactions.

## Discussion

This NBS program for PCD comprised a large cohort of 1,247,274 newborns, most of them were from eastern China. A total of 40 newborns were diagnosed with PCD, yielding a prevalence of PCD in Shanghai of 1:31,200. We further analyzed the clinical, biochemical, and genetic characteristics of patients with PCD, and investigated the correlation between biochemistry and different mutation types. Moreover, we identified 8 novel variants from the *SLC22A5* gene and predicted the protein structure effects on the new missense mutations. These findings expanded the PCD disease spectrum in Chinese patients and increased concerns about early diagnosis and therapy.

MS/MS is currently recognized as the most reliable and quick method for the early diagnosis of PCD by detecting the C0 level. However, C0 levels in infants shortly after birth reflect the carnitine levels of their mothers, because carnitine is transferred from the placenta to the fetus during pregnancy, which may lead to false-positive and false-negative diagnoses (El-Hattab et al., 2010; Magoulas and El-Hattab, 2012). Moreover, premature birth, mitochondrial diseases, insufficient intake, several organic acidemias and fatty acid oxidation defects, can lead to secondary carnitine deficiency (Lin et al., 2020). Therefore, a further differential diagnosis is required to rule out carnitine deficiency caused by these factors, so as to determine whether it is PCD. Notably, case 18 showed slightly decreased C0 levels at initial screening and recall (8.12 and 9.87 μmol/L, respectively) and harbored two pathogenic variants in the *SLC22A5* gene, so that he may be readily missed if the recall procedures are not strictly followed. In addition, some PCD patients with one mutation were identified in this study, which might be related to sequence technology since it cannot detect variants in the regulatory regions or deep introns, and large deletions or duplications. This is also a limitation of this study, especially in patients with severely low C0 levels, such as case 9, which might present an important undetected variant. Collectively, MS/MS combined with genetic testing become an effective strategy to avoid missed diagnoses, especially in newborns with C0 levels closing to the lower reference limit.

The *SLC22A5* gene is located on chromosome 5q31.1, containing 10 exons and three introns. So far, more than 150 disease-causing variants have been reported, most of which are missense mutations. The variant spectrum of *SLC22A5* differs in different races and regions. For instance, the mutation c.95A>G (p.N32S) is common in Faroe Islands (Rasmussen et al., 2014), c.136C>T (p.P46S) is common in the Texas state of United States (Li et al., 2010), c.454G>C (p.G152R) and c.760C>T (p.R254X) are common in Turkey, and c.1400C>G (p.S467C) is common in Japan (Koizumi et al., 1999; Kilic et al., 2012). In China, the variant spectrum of *SLC22A5* is also varied in different regions and provinces. A previous study found that c.1400C>G (p.S467C), c.51C>G (p.F17L), and c.760C > T (p.R254X) are common in Chinese patients (Lin et al., 2020). Among them, c.1400C>G (p.S467C) is the most common variant in Zhejiang, Jiangsu, and Henan provinces, and c.760C > T (p.R254X) is common in Taiwan (Tang et al., 2002; Lee et al., 2010; Sun et al., 2017; Zhou et al., 2019; Lin et al., 2020). In contrast to these reports, this study found that c.51C>G (p.F17L) and c.1400C>G (p.S467C) were the most common variants in PCD newborns, and the frequencies of these two mutations among the identified variants showed no significant difference, which indicated that these two variants might be hotspot mutations in eastern China. Furthermore, 8 novel variants were identified in this work: c.248G>A (p.R83H), c.361C>T (p.Q121X), c.384dupT (p.V129Cfs*9), c.407G>A (p.C136Y), c.494A>G (p.D165G), c.621G>T (p.Q207H), c.1133T>G (p.L378R), and c.1199G>A (p.R400H), expanding the variant spectrum of the *SLC22A5* gene. The possible pathogenicity and protein structure effect on the novel mutations predicted by the bioinformatic analysis indicate that these variants may be potential etiologies of PCD, and further functional research is required to evaluate the effects of these new mutations.

Consistent with previous report, early diagnosis and treatment have good outcomes for patients with PCD (Tang et al., 2002). Within our cohort, 70% of patients were asymptomatic, and 5% suffered from easy fatigue, mild left ventricular enlargement, and muscle weakness. Unfortunately, 25% of the patients were lost to follow-up, and the possible reason was poor treatment compliance because they might have no symptoms without treatment. In this study, c.760C > T (p.R254X) was the most common non-sense mutation, which can cause a premature termination codon at amino acid 254 and prevent OCTN2, leading to obvious clinical manifestations (Tang et al., 2002; Han et al., 2014; Frigeni et al., 2017). Four PCD patients with R254X mutation identified by NBS showed no obvious clinical manifestation after carnitine supplementation and dietary guidance. However, PCD patients with two R254X mutations diagnosed in the clinic presented muscle weakness and cardiomyopathy, those with R254X and other mutations suffered from heart failure, cardiomyopathy, hepatomegaly, diarrhea, feeding difficulties, fever, and even death (Han et al., 2014). Therefore, standardized medication instruction and targeted treatment adjustment are essential for Chinese PCD patients, while asymptomatic patients also need long-term follow-up and treatment to monitor their health conditions, highlighting the importance of NBS.

In order to gain further insight into the biochemistry and genotype correlation in PCD, we analyzed the C0 levels of different mutation types in the patients. Based on a previous report, PCD patients with non-sense mutations or frameshift mutations show extremely feeble OCTN2 activity, while those with missense mutations have partial residual OCTN2 activity (Kilic et al., 2012). Nowadays, the relationship between the C0 level and *SLC22A5* genotype in PCD patients identified by NBS is unclear (Zhang et al., 2019). However, it was found in this work that the primary screening and recalled C0 levels in patients with c.51C>G (p.F17L) and a non-sense mutation or frameshift mutation were lower than those with c.51C>G (p.F17L) and other missense mutations. All compound heterozygous PCD patients with c.51C>G (p.F17L) and a non-sense mutation or frameshift mutation have a C0 value of <5 μmol/L. The C0 level (1.03 μmol/L) of case 3 with c.396G>A (p.W132X) and c.1400C>G (p.S467C) was the lowest among all screened PCD patients, and was 15% of those with c.1400C>G (p.S467C) and other missense mutations (6.72 ± 1.16 μmol/L). In addition, 2 novel variants were identified from the non-sense or frameshift mutations, c.361C>T (p.Q121X) can result in a premature termination of translation and presented between transmembrane domains 1 and 2, c.384dupT can cause a frameshift of p. V129Cfs*9 at the first extracellular topological domain of OCTN2. Both patients with these two mutations had a C0 value of <5 μmol/L, which further suggested that non-sense or frameshift mutations might affect the C0 levels of PCD newborns more greatly.

## Conclusion

In conclusion, NBS *via* MS/MS was an efficient and reliable method for early diagnosis of PCD. The incidence of PCD in Shanghai was 1:31,200. Eight novel variants were identified in this study, greatly expanding the variant spectrum of *SLC22A5* gene. c.51C>G (p.F17L) and c.1400C>G (p.S467C) were the most common variants of newborns in Shanghai. The combination of biochemical and molecular analysis could effectively increase the diagnostic accuracy of PCD.

## Data Availability

The data presented in the study are deposited in the SRA database, accession numbers: SRR22420523-SRR22420530.
