# Supplementary material for: Clinical, biochemical, and molecular genetic characteristics of patients with primary carnitine deficiency identified by newborn screening in Shanghai, China
Source: Front Genet. 2022 Dec 8;13:1062715. doi: 10.3389/fgene.2022.1062715 (PMC9772520; doi:10.3389/fgene.2022.1062715)
Supplement: Supplementary file 1 [file Table1.DOCX]

**Supplementary table 1: Primer sequences of 10 exons of *SLC22A5* gene.**

| **Exon** | **Forward primer** | **Reverse primer** |
| --- | --- | --- |
| Exon-1 | GTCTTGGGTCGCCTGCTG | GAGCTCGGGTTCAAGGACC |
| Exon-2 | TACTCTCTGCCTGTCTCTCCTC | ACCAACTGAAATCAAGGGCCAG |
| Exon-3 | ATAGCATGGGCACTGTGAGAC | TAAGGTTCACCAGGAAGCTCTG |
| Exon-4 | CTCCCTAGCGCCATGAACTTA | TAGGGATTCATGGGTTGTTGCT |
| Exon-5 | CCAGGTTATTGCTGCGTGTG | TGTGAGCAGGGAGGACTTCA |
| Exon-6 | TAGAAACGTAACACTCCCCGAC | AGGCTTTGAATTTTGTCTGAGGC |
| Exon-7 | AGTAAGACGCAGGGTTACAGT | GAGACTCAGTGAAGACCCCAA |
| Exon-8 | ATAGCCCCTTCCCCCACAATA | GTACTTCCATCCCGTTGCTCT |
| Exon-9 | TCCTGGGAGCATAAAGGGGTA | TGGTGTCTGTGAGAGGGAGTT |
| Exon-10 | GTTTGTTTGGAGACTGGGAGG | TGCTAGAATTAGCCCAAGCTG |
