# Supplementary figures and images for: Clinical, biochemical, and molecular genetic characteristics of patients with primary carnitine deficiency identified by newborn screening in Shanghai, China
Source: Front Genet. 2022 Dec 8;13:1062715. doi: 10.3389/fgene.2022.1062715 (PMC9772520; doi:10.3389/fgene.2022.1062715)

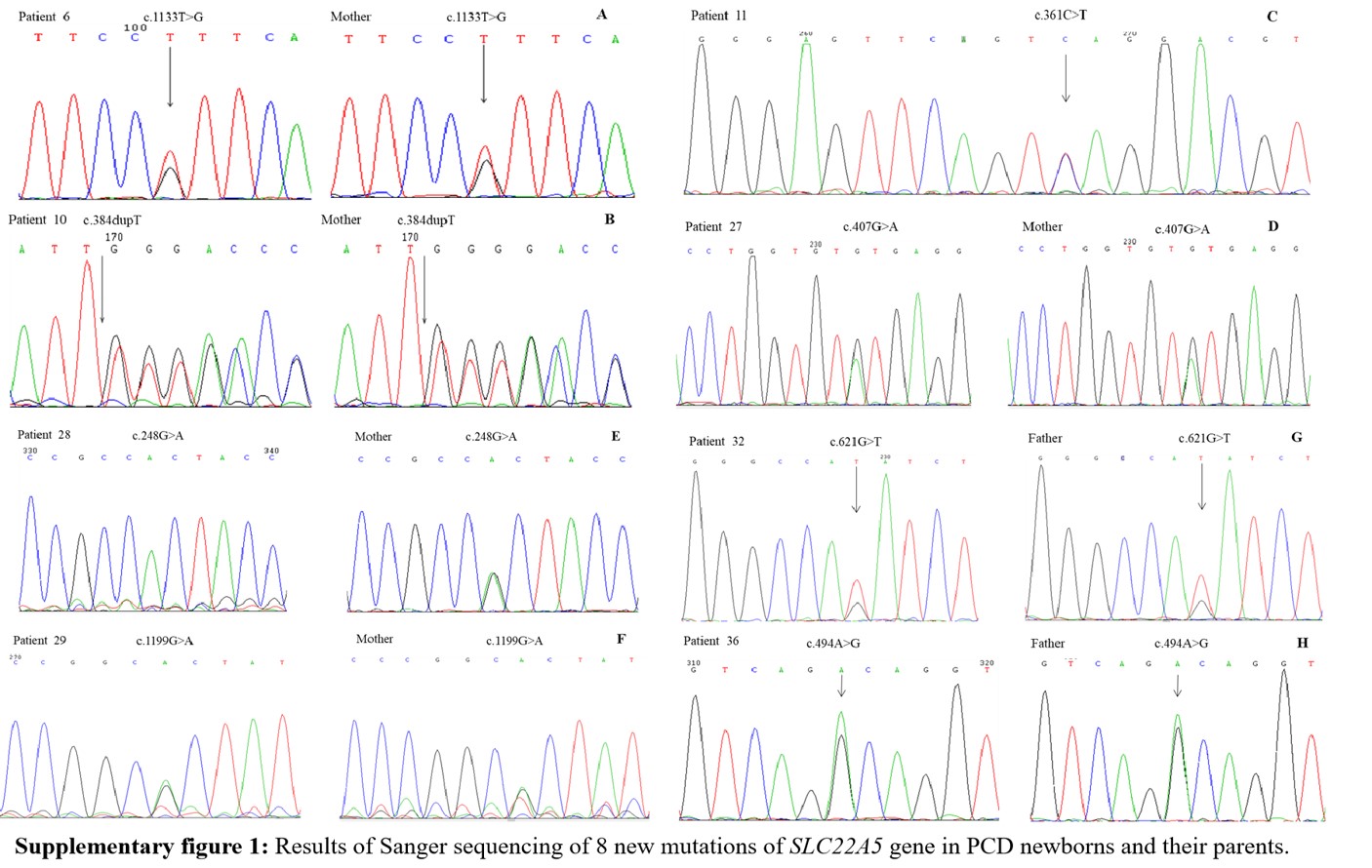

Supplement: Supplementary file 2 [file Image1.JPEG]

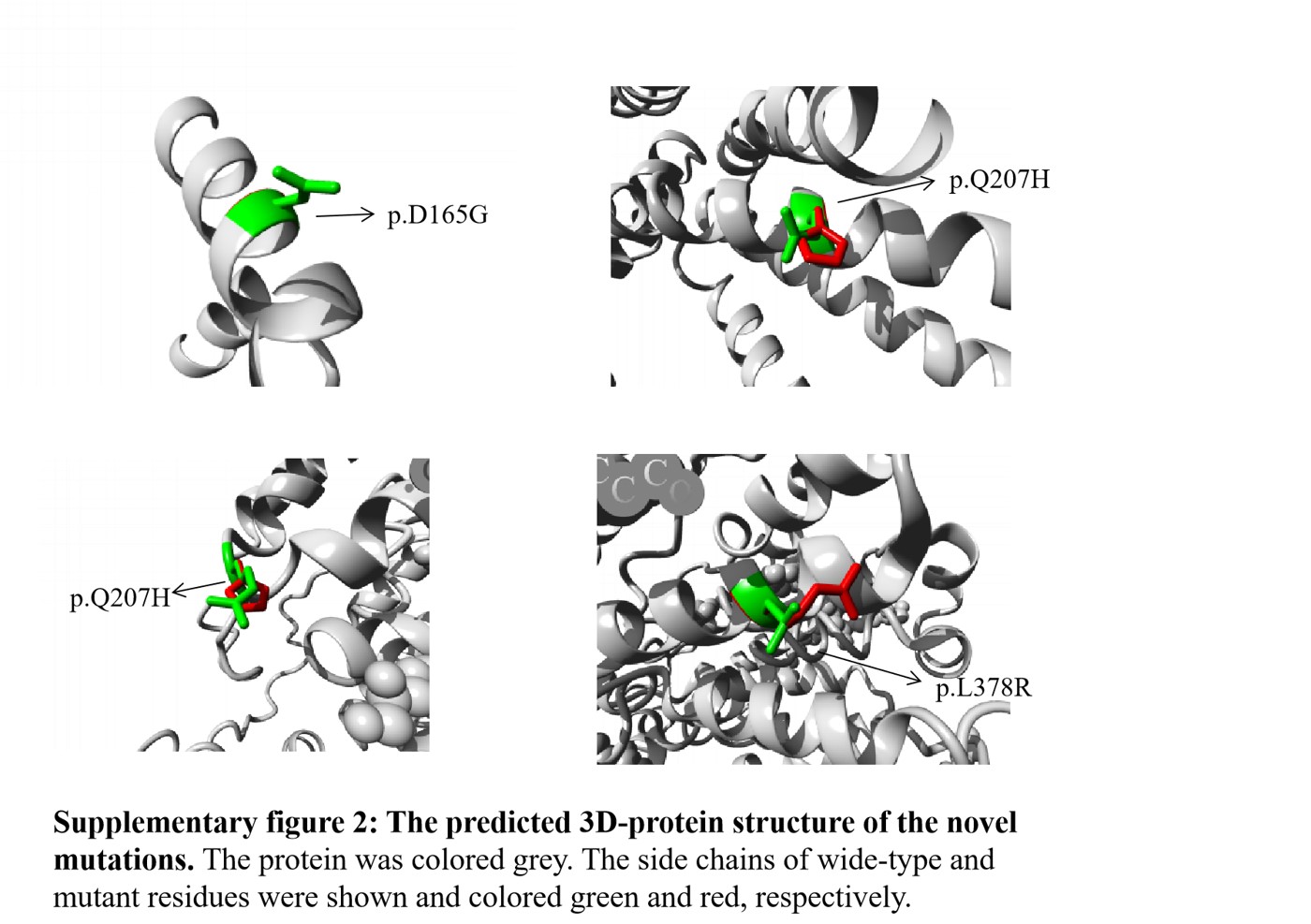

Supplement: Supplementary file 3 [file Image2.JPEG]
